# Applied Principles for Inclusive Practice in Neurodevelopmental Research: A Selection and Report of Illustrative Case Studies

**DOI:** 10.1007/s40474-025-00318-1

**Published:** 2025-02-17

**Authors:** Sue Fletcher-Watson, Holly Joseph, Laura Crane, Georgia Pavlopoulou, Steve Lukito, Eloise Funnell, Alyssa M. Alcorn, Catherine J. Crompton

**Affiliations:** 1 Salvesen Mindroom Research Centre, University of Edinburgh, Edinburgh, Scotland; 2 Institute of Education, University of Reading, Reading, England; 3 School of Education, University of Birmingham, Birmingham, England; 4 Group for Research in Relationships and NeuroDiversity- GRRAND, Department of Clinical, Educational and Health Psychology, University College London, London, England; 5 Anna Freud Centre, London, England; 6 Department of Child & Adolescent Psychiatry, Institute of Psychiatry, Psychology and Neuroscience, Kings College London, London, England; 7 School of Psychological Science, University of Bristol, Bristol, England

**Keywords:** Coproduction, Codesign, Inclusion, Neurodevelopment, Neurodiversity, Participatory methods, Case study

## Abstract

**Purpose of Review:**

Inclusive research practices are important for neurodevelopmental studies, facilitating the involvement of community members throughout the research process. Highlighting this value, we reiterate our previously proposed framework for inclusive research practice and present a selection of case studies showcasing successful implementation of inclusive approaches.

**Recent Findings:**

Across four invited case studies, authors illustrate how neurodivergent people can be effectively involved in research, providing meaningful input and shaping outcomes. Our report concludes that these case studies underscore the significance of building relationships, prioritizing community well-being, and considering diverse identities in neurodevelopmental research. We call for careful evaluation of the impact of inclusive practices on community representatives and advocate for enhanced reporting in academic journals, and use of online repositories to share the materials that support coproduction.

**Summary:**

Despite the recognized benefits, a lack of detailed reporting on inclusive methods poses a challenge for researchers. This report provides valuable insights for researchers aiming to instigate, establish or develop their inclusive practice.

## Introduction

Inclusive research takes place when members of the community who are the focus, intended beneficiaries or planned users of the research are involved in the process. It deploys participatory methods in tasks such as defining research questions, designing methods, collecting, analysing and interpreting data, and disseminating findings. Inclusive research can be delivered via various models which represent the degree of power, normally reserved to professional academic researchers, instead held by community representatives [1]. Even within the broad categories described in this previous article (Consultation, Partnership, Collaboration, Citizen Science and Leadership) multiple specific ways of doing participatory work are possible. For example, one type of Collaboration is co-design, where community representatives collaborate to set goals and design methods and / or materials for a research study, intervention or output. Co-production, another form of collaboration, suggests more integrated involvement in the entire pathway, from design to delivery and dissemination, with community members involved in data collection, analysis, interpretation and sharing of findings.

Whatever the chosen approach, inclusive practice in neurodevelopmental research is a moral imperative and has practical benefit. Both motivations primarily derive from the fact that neurodevelopmental diversity means that neurodivergent people,^1^ often having one or more neurodevelopmental diagnoses, experience the world in distinctive ways [2]. There is no guarantee that a given research team will include people who have lived experience of that neurotype, especially in the context of systemic barriers for neurodivergent academics [3]. Thus, it is usually necessary to be proactive in bringing community representatives into the research context, via inclusive practices. Involving neurodivergent people can help researchers meet their moral obligations, in relation to dismantling ableist beliefs and practices (often constructed within academia), and result in pragmatic benefits. These include improved data quality, effective recruitment, and translational impact, among others.

**Fig. 1:**
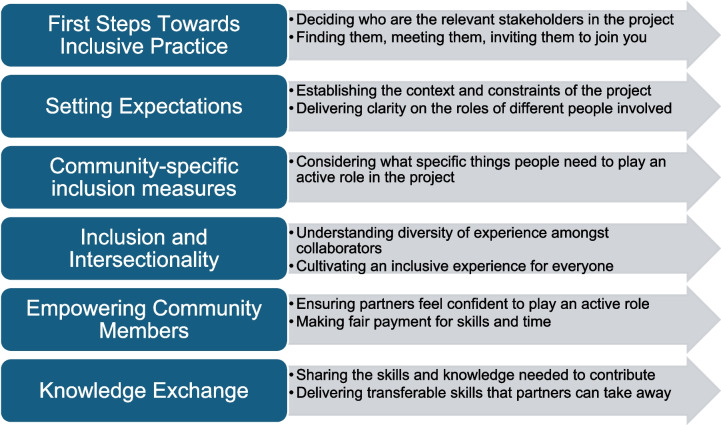
Elements of Inclusive Research Practice with Illustrative Researcher Actions

In Fletcher-Watson et al. [1] we presented six elements of inclusive research practice, illustrated above in Fig. 1. This figure includes non-exhaustive indicators of researcher actions within each element of the whole. We do not propose this as a definitive theoretical model of inclusive research, but instead as a useful reference or framework to support researchers focused on neurodevelopment to embed inclusive practices. It can be applied whatever type of participatory approach is being used.

A frequently-noted limitation when it comes to the development of inclusive practice is the difficulty of reporting inclusive methods. The literature abounds with discussions of why inclusion matters and principles of delivery but accounts of methods of inclusion are rare [4–6]. These are badly needed if researchers are going to be able to instigate and evolve their participatory approaches [7]. This is often because inclusive methods are normally only part of a project, and journal articles rarely provide space for these steps to be reported in any detail.

Here we respond to this limitation by inviting four case studies of inclusive neurodevelopmental research. The purpose of this report is to provide concrete examples of inclusive practice at more length than currently customary within academic papers, as a model for future brief reporting and a resource for learning.

## Methods

Given the established context in which inclusive methods are under-reported, often consigned to supplementary materials at best, or described in sparse detail, we were unable to draw examples from a review of the literature. Instead, we worked in professional networks to identify case studies of good practice, using the following criteria to guide our selection:

1. Inclusive practice relevant to the remit of the journal, namely ‘developmental’ (working with children, young people and / or families) and ‘disorders’ (inclusive of neurodivergent people).
2. Case studies led by researchers with established inclusive practice, who had developed their knowledge and skills to a high level.
3. Case studies working across multiple diagnostic groups, with groups having multiple diagnoses and / or with people having no diagnosis. We wanted to showcase projects that had wide applicability to researchers regardless of the population they are working with.
4. Case studies showcasing work across diverse settings and applications: fundamental research, healthcare, education and community.

Finally, while not a strict criterion, we limited ourselves to UK-based case studies to bypass the need to establish different local norms in terms of attitudes, diagnoses, services and so on for each case study. However we note the importance of inclusive practice everywhere in the world and the particular need for inclusive methods when researchers conduct research across national or cultural lines. Case studies were invited to be written with reference to our framework (Fig. 1) and provide a range of examples of how different research teams, in varying contexts, worked to embed inclusive practice.

## A Community-based Initiative with Young Families to Foster a Love of Reading, by Holly Joseph

### About our Project

Our community-based project aimed to foster a love of reading in families living in a low-income urban area. We know that children from less affluent families are much less likely than peers to receive a diagnosis of dyslexia [8]. It was therefore important to look beyond diagnosis and work within communities to support family literacy in an accessible and sustainable way. Through extensive consultations, three main activities were co-developed, delivered and evaluated: shared storybook reading sessions for parents and toddlers, a book club for parents, and a summer school for preschool children. Our project was a collaboration between university researchers, community researchers (local people who are trained by the university to conduct research), local parents, and community leaders.

### First Steps Towards Inclusive Practice

Our approach was informed by the concept of family and intergenerational literacy [9], which emphasises the influence of parental literacy on children’s engagement with reading, so parents were our key target group. We capitalised on existing relationships formed through participatory research projects within the university [10] to set up meetings with community leaders: local councillors, teachers, charity workers, youth workers, social workers, librarians, and community centre leaders. These conversations helped us to understand community perceptions, revealing that past government initiatives had often been viewed as patronising or out of touch, and this informed how we then approached families.

### Setting Expectations

Establishing expectations was a significant challenge due to differing communication styles of researchers and community members. For example, many parents preferred texting over email and were initially hesitant to engage with formal information sheets and consent forms. Flexibility was therefore essential in the early stages of relationship-building. Community researchers, already embedded in local groups with established social networks, played a key role in maintaining this flexibility. Once trust was built, it became possible for parents and researchers to communicate clearly and effectively, ensuring, for example, that we followed agreed institutional and research ethics procedures while respecting the preferences of participating families.

### Community-Specific Inclusion Measures

Ensuring the research design was tailored to community needs was our primary objective. One key element was the selection of meeting and event locations. Initial plans to involve local nursery and primary schools were revised when parents expressed negative associations with these settings. Instead, locations that parents found welcoming and safe were chosen (e.g. cafés and community centres). Providing refreshments, compensating for time, and offering free childcare at all meetings and activities were essential inclusion measures. These efforts helped reduce barriers to participation and demonstrated respect for the parents' time and commitment. Clear and respectful communication was also critical to avoid the perceived condescension of past initiatives.

### Inclusion and Intersectionality

Many parents and community members faced multiple forms of marginalisation, creating barriers to self-advocacy. All participating parents were women, some of whom had experienced trauma, which in turn affected their wellbeing and engagement with educational activities. Conversations revealed that many had been met with barriers to learning in school, particularly related to attention and literacy. As well as adapting any written information to increase accessibility, we addressed these multiple intersecting identities by listening, not making assumptions, and focusing on supporting families holistically, rather than through the lens of diagnostic categories, gender or trauma. We deliberately didn’t ask for diagnostic status, partly because of known underdiagnosis of literacy difficulties in low-income families, but also to resist a categorisation which does not reflect the complexity of intersecting identities.

### Empowering Community Members

Employing and training people as community researchers was a powerful part of the project. Our community researchers were also local parents which meant that they could build on existing strong relationships, sharing their stories and facilitating contact with other community members. Community researchers, selected for their diverse expertise and experience, were involved in designing sessions, recruiting families, conducting interviews, and contributing to data analysis. In addition, both parents and community researchers helped disseminate findings to non-academic audiences, including the media, the local council, and the local Member of Parliament. They were also recognised in research bids, co-authored papers, and were nominated for research awards. This acknowledgment underscored the project’s commitment to genuine community involvement and empowerment.

### Knowledge Exchange

The project allowed the university researchers to understand the experiences and stories of local parents that feed into children’s early literacy experiences. Because the nature of the project demanded their attention and time in the community, a deeper understanding of the context was gained, and they will take this to future projects. The community researchers learnt research skills and gained experience engaging with the media and policymakers. Local parents reported multiple benefits from participating in our project including forming new friendships, rediscovering reading, and one parent went back to college to gain qualifications and has recently been offered a place at university.

## Inclusive Research on Post-diagnostic Support for Autistic Young People, by Laura Crane

### About our Project

Our team of academics partnered with a charity who wanted to design and deliver a post-diagnostic peer support programme for autistic young people. The charity wanted the programme to be co-designed with autistic young people and underpinned by collaborative research with this group. The charity’s brief was that the research needed to (1) review existing research to inform programme content, (2) identify how to ensure that the programme was inclusive of a broad range of autistic young people, and (3) evaluate the co-design and pilot of the programme. Within this broad brief, there was flexibility in the design of the research, and a summary of the work is available in Redmayne et al. [11].

### First Steps towards Inclusive Practice

The charity we partnered with is not Autistic-led. However, the charity runs a Youth Network, comprising autistic young people (16–25 years), who meaningfully contribute to the charity’s work and are financially compensated for their involvement. The charity advertised for six members of the network to collaborate on different elements of the research. Collaborators were purposefully recruited so that their backgrounds/expertise were directly relevant. For example, we specifically sought collaborators who identified as having at least one minoritised identity since our research was examining how to make the peer support programme maximally inclusive (see [12]).

### Setting Expectations

The charity had clear expectations around involvement of their young people, in terms of how roles were advertised and undertaken. The academics ensured that the charity’s procedures and policies were followed, so that collaborators were aware of the funding available for the role, the time commitment involved, and the format by which they could contribute. This approach was felt to foster a sense of familiarity for the collaborators, and a staff member from the charity provided oversight and support throughout.

### Community-Specific Inclusion Measures

Together with our collaborators, we identified three autism-specific inclusion measures that underpinned the success of the research (see [13]). First ‘maximising success through preparation’, e.g., taking time to develop clear rules of engagement for online meetings and sticking to these. Second, ‘facilitating effective and respectful communication’, e.g., through explicitly encouraging respect for diverse opinions. Finally, ‘empowering meaningful collaboration’, e.g., by giving agency over the topics to be addressed, and in what order.

### Inclusion and Intersectionality

Conscious of the diverse range of collaborators involved in the project, with various intersecting identities, every member of the team provided a one-page profile that included information about the person, such as how they could best be supported during the research process. Profiles were shared at the start of the collaboration and, crucially, were acted upon. For example, one of our collaborators required a British Sign Language (BSL) interpreter, so we ensured that one was present at all meetings. Guidance around best practice for BSL interpretation in online meetings was also followed.

### Empowering Community Members

Our collaborators had varied experience of research, so we spent time learning about their backgrounds, and what they hoped to gain from taking part. We aimed to support the development of collaborators’ research literacy through activities such as running ‘journal club’ discussions for the sub-group involved in our literature review. Specifically, we introduced them to quantitative and qualitative journal articles. We navigated them through these research reports, before engaging in structured discussions about the quality of the work and the implications for practice (see [13]).

### Knowledge Exchange

Upskilling all involved in the project was a significant outcome of the work. For example, the collaborators benefitted from developing their research knowledge and expertise, and they gained important transferable skills to take into education and/or employment. The charity benefitted from working alongside academic researchers, learning how high-quality research design could inform the services they offered, complementing the insights gained from the autistic young people. Finally, the academics benefitted from modelling the charity’s existing inclusive working practices and gained valuable insights from the collaborators regarding aspects of research design and delivery to inform future projects.

## Participatory Approaches to Improve Translational Neuroscience by Georgia Pavlopoulou, Steve Lukito & Eloise Funnell

### About our Project

The Regulating Emotion – Strengthening Adolescent Resilience (RE-STAR) is a Medical Research Council/ UK Research & Innovation-funded multi-disciplinary research programme that aims to reduce risk of depression in secondary school students with a diagnosis of autism and/or ADHD. From the start, the programme included a group of neurodivergent young people as advisors. During the first year, we ran a series of online meetings to define together and activate mechanisms that would allow them to fully integrate within RE-STAR team as co-researchers [14]. We are now entering the third year of our collaborative partnership, and the youth co-research group is firmly embedded within all RE-STAR work packages.

### First Steps towards Inclusive Practice

In 2023, funding was awarded to an academic and two youth-co-researchers from RE-STAR to establish a second co-researcher group of young people, co-designing and co-delivering the optimal electroencephalography (EEG) lab setting for young neurodivergent participants. Youth co-researchers were recruited through open calls to join the team in roles including EEG study designers, data collectors, systematic reviewers, and data analysts.

### Setting Expectations

The collaboration was built on our existing co-produced duty of care protocol and a set of principles for conducting participatory research [14], which include among others: shared passion for the study,team commitment to the goal of improving the life chances of neurodivergent young people; trust and confidence founded on transparent communication among team members.

We aimed to co-produce an EEG study protocol in the first three months, and to execute the study plan subsequently. The organisation of the programme, which incorporated comments and feedback from the youth co-researchers, consisted of weekly planning meetings – and later, monthly reviews – reflection sessions, and regular journalling. These were all clarified in a weekly/monthly planner and briefing document emailed to applicants. One-to-one meetings were arranged to discuss further questions and provide additional reassurance for co-researchers with no scientific background.

Youth co-researchers were expected to attend a set of meetings, or watch their recordings when unable to attend. Missing attendance did not impact on remuneration, and opportunities for one-to-one catch-up sessions were provided for those who missed more than one meeting in succession.

### Community-Specific Inclusion Measures

We used a mixture of modalities to facilitate communication between academic and youth co-researchers. Co-researchers provided regular verbal and written feedback using Padlet, email and Zoom meetings which supported us to respond to young peoples’ preferences. Training needs were recorded, and training plans were co-developed. Induction sessions were mostly arranged online after work hours, allowing the youth co-researchers to participate from the comfort of their familiar surroundings. Data collection sessions were at the weekend, and organised so that young co-researchers could be involved with several sessions within a single day trip.

### Inclusion and Intersectionality

In addition to being neurodivergent, some of the co-researchers have mental health and physical disabilities. Specific travel conditions (e.g., quiet trains, travel with supportive partner) were accommodated, as well as non-speaking ways of participating during online meetings. Respecting, and monitoring for any change in people’s preference for expressing their identities, e.g. via their pronouns, is part of our day-to-day work. An ongoing challenge has been to recruit young co-researchers from underrepresented ethnic groups. We have been networking with local charities to understand better how to overcome this during advertisement and recruitment.

### Empowering Community Members

Co-researchers were reimbursed for their training, preparation and meeting time. The academic team ensured that youth co-researchers have been consistently offered co-authorship opportunities and public speaking opportunities. Co-researchers routinely share the conference stage as equal contributors (e.g. EUNETHYDIS 2022 and ITAKOM 2023) – where talk about science and their own experience are interwoven. The latter entailed rigorous written and visual task planning, collaborative iterations on creation of slides and rehearsals in subgroups. Peer support and academic support before, during and after the event was made available and personalised to the needs of each team member.

### Knowledge Exchange

No prior experience was necessary for co-researchers, and induction training included online sessions on quantitative research, EEG methods and research ethics as well as in-person session for EEG data collection. This enabled youth co-researchers to be fully involved in formulating study rationale, research question and hypothesis, and in decision-making over study design, measures, and tasks.

Our young co-researchers have led a series of public events to share our methods and findings. One wrote a blog entry about their participatory research experience in the institutional “King’s Engaged Researchers” online forum [15], while another co-first authored a scientific poster outlining our research plan in a conference [16]. With other participatory research groups, we have recently produced an exhibition titled “Experts by Experience: Who Knows Best?” and a manifesto of participatory research.

## Designing Resources for Whole-class Learning About Neurodiversity in Mainstream Primary Schools, by Alyssa M. Alcorn

### About our Project

The Learning About Neurodiversity at School (LEANS) project designed and evaluated a teacher-delivered, whole-class programme to educate children aged 8–11 about neurodiversity and neurodivergence and foster more inclusive attitudes and actions [17–19]. LEANS’ research leadership group was neurodiverse, including academic researchers, neurodivergent community members, and a charity partner. The funder’s remit was unusually open, committing us to create a neurodiversity resource for UK mainstream primary schools (i.e. schools that are not specialised provision for disabled pupils)—and to develop its goals, content, format, and delivery through participatory work with “adult experts”. This case study focuses on the initial design process.

### First Steps towards Inclusive Practice

We first “designed the design”, outlining seven iterative cycles of idea generation tasks and decision-making meetings. We developed a pre-specified Participatory Design Team (hereafter, the design team) member role, similar to a job description, before approaching prospective members. It explained responsibilities, timeline, and pay as well as the design phase and team’s fit within the larger project. Crucially, it stressed non-negotiable project constraints, such as age group, and that prospective team members had to be willing to work within the currently-funded remit.

Both lived experience of neurodivergence and work in education settings were equally essential for the role. We advertised primarily through the researchers’ educational contacts, inviting applications for the team. Researchers independently scored design team candidates, seeking to include a range of lived and professional experiences, to maximise geographic diversity, and to represent other diverse experiences as far as possible (e.g. social/economic, cultural, religious, LGBT + communities). We invited eight members to form the final design team.

### Setting Expectations

Expectations were already set to some degree by the team member role description. Before work began, we also provided a design handbook documenting background information, planned process and timeline, people’s roles, and practicalities (e.g. use of online platforms, payment). It set out key rules/procedures such as the quorum for binding decisions, and complaints processes. All researchers and designers were additionally required to sign a conduct agreement.

### Community-Specific Inclusion Measures

With neurodiverse research and design teams, our inclusion measures focused on 1) clear and consistent communications about plans and expectations, and 2) offering flexibility where possible (and being clear if and when this was *not* possible). Examples of clear communication included the aforementioned design team handbook, setting and adhering to conventions for labelling deadlines and actions (e.g. in team e-mails), and publicising required decisions ahead of live meetings. Examples of flexibility included on-demand technical help, asynchronous tasks within each design cycle, and dedicated channels for offline follow-up/additions to live meetings. All options were available to everyone as standard.

### Inclusion and Intersectionality

The final team was highly diverse in experience, location, and types of schools/communities, but not in cultures/ethnicities, highlighting an area for improvement. Partly in response to this, a later dedicated consultation study recruited experts by experience (i.e. people with minority identities) to shape our visual and written representation of diversity (gender, ethnicity, disability, culture) in project materials.

Our all-online design process, implemented out of necessity due to 2020 lockdowns, was ultimately beneficial and popular. It facilitated a more diverse, geographically dispersed team (including from remote/rural schools) and was more logistically manageable for people with caring responsibilities. Working online also removed additional burden from team members, associated with travel and meeting in an unfamiliar place, and was advantageous to our budget.

### Empowering Community Members

This case study describes the first phase of a participatory design project, in which an educator design team contributed to initial development of a classroom resource. We were up-front with team members that development was iterative, and the resource would continue evolving in later phases due to planned consultation studies [20] and other stakeholder input. Evaluation, data analysis, and reporting were outwith the design team's role. Participatory design, rather than full co-design, was the right choice for our resourcing and aims, and design team members were empowered to act within that framework.

LEANS planned the extent and number of design team roles based on our budget to pay contributors an hourly rate for their time and experience. In every design cycle, team contributions were creative and plentiful, generating extensive discussion. Between finite budget and tight timing, we perceived (at the planning stage) a major risk of becoming ‘stuck’ on early design issues, leaving LEANS with no design team involvement at later stages. For this reason, the design process sought democracy where possible, but researchers held final decision-making power to ensure the overall process kept moving, even where we did not reach consensus.

### Knowledge Exchange

The greatest flow of knowledge was from the design team to the researchers, sharing insider information about professionals’ needs and tasks in primary school environments, for example around lesson-planning, current inclusion practices, parent interactions, and sources of conflict. Team discussions revealed huge variability across school systems and individual practices, highlighting the importance of including content to explicitly guide local decisions about the programme—but also providing a space for knowledge-sharing between peers within the team. Design team members also highlighted conflicts between research and school practices. For example, talking about “potential harms” (common language in ethics forms and participant information) as scary and disproportionate, versus using the familiar language of safety and risk management. This process positioned design team members clearly as experts and the researchers primarily in a learning and synthesising role. Design team members were credited, according to their preference, in all released materials and acknowledged in publications. They have frequently contributed to public-facing events such as the resource launch, and many continued to be involved in later research developments.

## Final Summary

These case studies reflect excellence in inclusive research practice in a range of projects. In different ways, each of them effectively brings community representatives into the research process, as decision-makers and influencers, not merely as passive subjects. Here we draw out some common reflections towards our two goals of improving reporting and facilitating learning in this area.

Notably, all of the teams have established inclusive practice as a norm, and it is clear that their capacity to build on pre-existing relationships was advantageous. When researchers have a positive reputation within the relevant community this can deliver a strong response to open recruitment calls and sometimes result in invitations to join community-led projects. Experience with community co-working can also help when it comes to navigating challenging boundaries – for example when institutional risk aversion and governance standards (e.g. informed consent models) conflict with a desire to share control more equally [21]. Readers for whom inclusive practice is brand new should make a gentle start, and focus on relationship-building and modest, short-term gains. For example, helping to identify priority questions for a new project via a focus group. Early work can then provide foundations for more ambitious collaboration in the future –it is clear that each project represented here is part of a longer timeline of past investment and future planning.

Inclusive practices prioritise comfort and wellbeing of community members as a matter of course, though researchers may have to confront some productive discomfort. Each case study gives examples of how researchers considered the past experiences and current needs of their community partners, not just in order to facilitate effective involvement, but to make the process manageable, rewarding and enjoyable. In contrast, research data collection – while it obviously aims to avoid harm or distress – is less concerned with enjoyment. Success in this regard is a key part of retaining community partners, not just for the life of the project but building long-term capacity as well, as demonstrated in the RE-STAR project evolution.

Projects reported here balance varying degrees of recognition and responsibility. Community partners had different levels of decision-making power and are acknowledged proportionately. Importantly, we reject the notion that good inclusive practice *always* means maximising community partner power [22]. In some cases, closely-defined roles can be more achievable for partners with limited time to contribute and balancing other responsibilities. Knowing that the researchers bear ultimate accountability can be liberating, and minimises community partners’ reputational risk. Honesty about the nature of the community roles and their decision-making power is a crucial part of allowing different research teams with varying budgets to engage with relevant communities, even when resource is constrained.

These projects give due consideration to the multiple identities of their community partners, both in terms of ensuring respectful day-to-day communication, and considering relevance to the project goals. It’s important however to note that the concept of “representativeness” as it is understood in quantitative research has limited utility here.^2^ When working with a group of a dozen or so advisors, we simply can’t claim to “represent” broader communities – certainly not in the sense in which this is used when talking about study samples. Instead, we should strive for diversity, such that collaborators bring a range of perspectives that can collectively improve the outcomes of research through variety in ideas, and perhaps also a degree of constructive friction [23]. Targeted inclusion of under-represented groups can push back against marginalisation [24].

Evaluating the success of inclusion measures and the degree of knowledge exchange and empowerment can be part of a continuous quality improvement process. In the projects described here, teams engaged in reflective discussions and feedback activities of various kinds but more formal quantification, reporting of benefits and identification of improvements, remains rare. It is unclear what ideal practice looks like in this regard [25]. Some kinds of measures could devalue community contributions, by positioning them almost as recipients of an “empowerment intervention” rather than valued, expert collaborators. Exit interviews or conscious reflective discussions may be more appropriate than surveys or feedback forms.

Reporting on participatory methodologies continues to be challenging in the absence of space to do so in most journals where neurodevelopmental research is published [26]. Making reporting compulsory can build trust and deliver benefits [5], but also cause problems if this puts pressure on authors to share personal aspects of their identity [4]. Meta-researchers have called on journal editors to provide additional space to report community involvement [6] and, in an age where most journals are accessed digitally there is no reason not to do so. In the meantime, we call on authors to use supplements to provide detailed reporting on involvement methods, and to consider sharing their materials on open science platforms, as in the case of this package of materials designed to facilitate recruitment of community representatives [27]. The authors invited to share case studies for this article found it relatively straightforward to follow our proposed reporting framework, which may prove useful as a model more generally.

In conclusion, these case studies provide exemplars which we hope researchers can use to motivate and evolve their participatory research practice. At the same time, they expose challenges in the field and illustrate why inclusive research is both difficult and necessarily bespoke to the population and topic under consideration. We encourage readers to take steps towards greater power-sharing with neurodivergent people in pursuit of research that is respectful, excellent and useful.

## Data Availability

No datasets were generated or analysed during the current study.

## Footnotes

1 We use this term to collectively describe a number of people who are often the focus of neurodevelopmental research, including people with relevant clinical diagnoses (e.g. ADHD, autism, developmental language disorder, developmental co-ordination disorder) or learning difficulties (e.g. dyslexia, dyscalculia, working-memory problems). The term is inclusive of people who fit these descriptions but whose needs have not been formally identified or labelled, people with multiple such diagnoses, and people whose profile of needs is divergent but lacks a classification.

2 In quantitative research, representativeness is usually used to refer to whether a sample of people have the same demographic properties as the wider population from which they were drawn – e.g. a sample of people with migraine would be majority-female. In qualitative research, representativeness instead can refer to whether the findings from an analysis accurately represent the underlying voices of the participants, directly assessed via measures such as member checking [28].
